# Genome-Based Comparison of *Clostridioides difficile*: Average Amino Acid Identity Analysis of Core Genomes

**DOI:** 10.1007/s00248-018-1155-7

**Published:** 2018-02-14

**Authors:** Adriana Cabal, Se-Ran Jun, Piroon Jenjaroenpun, Visanu Wanchai, Intawat Nookaew, Thidathip Wongsurawat, Mary J. Burgess, Atul Kothari, Trudy M. Wassenaar, David W. Ussery

**Affiliations:** 1 Molecular Microbiology and Genomics Consultants, Tannenstrasse 7, 55576 Zotzenheim, Germany; 2 Arkansas Center for Genomic Epidemiology and Medicine, Department of Biomedical Informatics, University of Arkansas for Medical Sciences, 4301 W. Markham Str., Slot 782, Little Rock, AR 72205 USA; 3 Division of Infectious Diseases, University of Arkansas for Medical Sciences, Little Rock, AR 72205 USA

**Keywords:** *C. difficile*, AAI, MLST, Community-acquired infections, Comparative genomics

## Abstract

Infections due to *Clostridioides difficile* (previously known as *Clostridium difficil*e) are a major problem in hospitals, where cases can be caused by community-acquired strains as well as by nosocomial spread. Whole genome sequences from clinical samples contain a lot of information but that needs to be analyzed and compared in such a way that the outcome is useful for clinicians or epidemiologists. Here, we compare 663 public available complete genome sequences of *C. difficile* using average amino acid identity (AAI) scores. This analysis revealed that most of these genomes (640, 96.5%) clearly belong to the same species, while the remaining 23 genomes produce four distinct clusters within the *Clostridioides* genus. The main *C. difficile* cluster can be further divided into sub-clusters, depending on the chosen cutoff. We demonstrate that MLST, either based on partial or full gene-length, results in biased estimates of genetic differences and does not capture the true degree of similarity or differences of complete genomes. Presence of genes coding for *C. difficile* toxins A and B (ToxA/B), as well as the binary *C. difficile* toxin (CDT), was deduced from their unique PfamA domain architectures. Out of the 663 *C. difficile* genomes, 535 (80.7%) contained at least one copy of ToxA or ToxB, while these genes were missing from 128 genomes. Although some clusters were enriched for toxin presence, these genes are variably present in a given genetic background. The CDT genes were found in 191 genomes, which were restricted to a few clusters only, and only one cluster lacked the toxin A/B genes consistently. A total of 310 genomes contained ToxA/B without CDT (47%). Further, published metagenomic data from stools were used to assess the presence of *C. difficile* sequences in blinded cases of *C. difficile* infection (CDI) and controls, to test if metagenomic analysis is sensitive enough to detect the pathogen, and to establish strain relationships between cases from the same hospital. We conclude that metagenomics can contribute to the identification of CDI and can assist in characterization of the most probable causative strain in CDI patients.

**Electronic supplementary material:**

The online version of this article (10.1007/s00248-018-1155-7) contains supplementary material, which is available to authorized users.

## Introduction

*Clostridioides difficile* (*C. difficile*) is a Gram-positive, anaerobic bacillus that is responsible for pseudomembranous colitis; it is also a common cause of nosocomial diarrhea, conditions whose morbidity and mortality have dramatically increased in the past decade [1–3]. Nosocomial infections caused by *C. difficile* are a recurrent problem and increasingly young individuals are recognized as being at high risk, in contrast with the historical *C. difficile* incidence trends, which pointed to elderly hospitalized patients mainly [4]. In the traditional view, hospitalized patients are exposed to the spores of *C. difficile* by direct contact with medical staff, via contaminated utensils or from the hospital environment, although food can also be involved in the transmission process [5]. In patients with an effective immune response, colonization of the gut by *C. difficile* can occur without the presentation of clinical signs. However, in individuals with a history of repeated exposure to certain antibiotics, those who are immunocompromised or suffer from underlying enteric diseases, *C. difficile* can dramatically increase in numbers [6]. This has knock-on effects on the gut microbiome and results in a disbalance of other bacterial species (dysbiosis), resulting in a semi-permanently altered gut micro-environment. Once this condition is established, treatment becomes very difficult. In such cases, a fecal transplant may be the only option, which has shown efficacy rates as high as 90% [7, 8].

The taxonomic description of the genus *Clostridium*, to which *C. difficile* used to belong, has undergone multiple revisions over the years. The genus expanded and was split up again, resulting in some confusion about its members. There have been at least 240 bacterial species that, at some time in the past, were accepted as a member of *Clostridium*. In 1994, a major revision of the genus was proposed, which moved a number of its members to novel genera [9]. This proposal was based on phylogenetic analysis of 16S rRNA sequences and was later backed up by phylogeny of a selection of protein genes [10]. Currently, there are 71 recognized species belonging to the genus *Clostridium* sensu stricto [11] whose type strain is *C. butyricum*. The species *C. difficile* is no longer part of this genus, as it was placed in the novel genus *Clostridioides*, together with *C. mangenotii* [9, 12].

The main virulence determinants of *C. difficile* are toxin A (ToxA) and toxin B (ToxB), which are encoded on pathogenicity locus (PaLoc) by *tcd*A and *tcd*B genes, respectively, together with three regulatory genes *tcd*C, *tcd*E, and *tcd*R [13]. It has been suggested that PaLoc is a mobile element [14], in which case transfer of the complete locus can convert a non-toxigenic strain into a toxigenic one [15]. In addition, a third toxin may be present, known as binary toxin or CDT (short for *C. difficile* toxin, not to be confused with the cytolethal distending toxin of Gram-negative bacteria). This toxin is encoded by *cdt*A and *cdt*B genes and belongs to the Iota-family of toxins to which also *Clostridium perfringens* toxin belongs [16], although the relevance of CDT in clinical disease is still discussed [17].

Genetic differentiation of *C. difficile* strains is important to identify possible nosocomial outbreaks, in which multiple patients are infected by a single strain. A common method for genetic differentiation is multilocus sequence typing (MLST). By this method, *C. difficile* strains have been classified into six phylogenetically different clades (clades 1 to 5 and C1) [13], although Clade C1 is not defined in the MLST database collected at the University of Oxford, UK. These clades may contain both pathogenic and non-pathogenic strains, but the vast majority of strains produce one or more toxins [13]. This suggests that MLST may not be an ideal approach to highlight differences among the toxin gene repertoire of the isolates under study.

Technical developments now allow routinely sequencing whole genome sequences (WGS), instead of a limited number of gene fragments only. A large number of WGS from *C. difficile* strains are already available in the public domain and these sequences have been useful in multiple ways. WGS characterization has been helpful in the study of *C. difficile* infections (CDI) [18], and it identified the true genetic diversity that exists among *C. difficile* isolates, which was originally assessed by identification of single-nucleotide variants (SNVs) within a limited set of known genes only [5]. In addition, WGS has assisted epidemiological investigations, as it is superior in identifying CDI transmission sources, in particular in patients with recurrent CDI infections [19–21]. By means of WGS, it was recently shown that most cases of CDI in hospitalized patients are due to endogenous strains carried by the patients who were asymptomatic prior to their hospitalization, while in the hospital, exposure to high doses of antibiotics may favor the growth of the bacteria, resulting in symptomatic CDI [22]. In such a scenario, even non-toxigenic strains can pose a risk, since they can acquire the PaLoc by horizontal gene transfer, carry alternative virulence genes, or result in the condition of dysbiosis [15, 23]. WGS has further been applied to assess the effectiveness of fecal transplants in severe CDI cases [7]. Lastly, metagenomics, in which all DNA present in a (clinical) sample is being assessed, is increasingly being applied to fecal analysis and may become a commonly applied technique in medical microbiology in the near future. Its main advantage is the identification of microorganisms without the need of culture, saving time in an outbreak investigation [24].

WGS results in a lot more data than what is assessed by SNP-based analysis or MLST. Since complete proteomes can be predicted, these can be subjected to average aminoacid identity (AAI) analysis, a method that compares all conserved protein-coding genes present in a given set of genomes, clustering strains into groups that sharing more than 95% AAI [25, 26]. This method has proven to have higher resolution power at the species level than comparison of 16S rRNA or MLST, since it assesses a far larger fraction of the genome [24].

Here, we compared all available *C. difficile* genomes by AAI analysis based on WGS data, starting with a comparison of taxonomic type strains of Firmicutes. The aims were to (1) determine if AAI analysis can confirm the status of *C. difficile* as a unique species; (2) assess if AAI produces groupings within the species that are of clinical relevance, to accurately identify pathogenic strains. For this second aim, the findings were compared to presence or absence of the toxin genes in different strains. As aim (3), we investigated whether metagenomics can identify CDI together with its associated strain (s), through a detailed analysis of published whole shotgun metagenomic sequences.

## Methodology

### Average Amino Acid Identity Analysis

AAI analysis was performed as previously described [27] and briefly summarized here. Amino acid sequences of all proteins from the analyzed genomes were extracted from their original GenBank accessions. AAI analysis was then carried out for every possible pair of genomes. The conserved reciprocal best match for each protein from each genome pair was first identified using UBLAST [28] with a cutoff of 30% sequence identity and a required minimum of 70% alignment length of the query sequence. For each pair of genomes, the average amino acid identity was then calculated based on the identities of all conserved reciprocal best matches, a calculation that is not always symmetrical. In such cases, the average of the two AAI values was assigned to each pair of genomes. Genomic clusters were then generated from the AAI values with a default cutoff of 95% (unless stated otherwise), meaning that members from different clusters cannot share more than 95% AAI identity, and members within a cluster have paths consisting of edges connecting isolates with AAI > 95%. The exact way how this clustering was done is described elsewhere [25, 26].

The first AAI tree was produced of all 25 taxonomically valid type strains of Firmicutes for which whole-genome sequences were available at the time of analysis. The list of type strains was obtained from the Names4Life website (www.namesforlife.com, accessed on 14 March 2017). The pairwise comparisons used to calculate AAI values for this set of genomes involved between 236 and 1766 genes (666 genes on average). The AAI tree was built with BIONJ [29] to dissimilarities of AAI values (100% minus AAI).

For comparison of all Clostridia members, the GenBank database was accessed on February 12, 2017, and all available complete genomes and chromosomes (*n* = 234) within the Clostridia class were downloaded. This selection was restricted to completely sequenced genomes and included 8 genomes from *C. difficile.* The AAI values of the Clostridia selection involved compared between 131 and 5017 genes (955 genes on average).

A third AAI tree was built using all 663 *C. difficile* genomes that had genome quality scores > 0.8 as defined elsewhere [30], this time including complete genomes as well as draft genome sequences available at the time of analysis. The dataset contained 653 sequences described as obtained from *C. difficile*, completed with 10 genomes with no species designation but presumed to be *C. difficile* based on gANI (genome-wide average nucleotide identity) [31]. Two genomes of *Clostridioides mangenotii* (GCA_000498755 and GCA_000687955) were added to serve as an outgroup. A tree that contains over 600 branches would be hard to read, and since many genomes are extremely similar, the tree would end in many very short branches. For graphical representation of such a large dataset, branches that contained identical or highly similar members were collapsed. We attempted to define a suitable cutoff for such a collapse by varying the required percentage of similarity within this dataset as described in the results. Bootstrap values were calculated conceptually similar to alignment-based trees: among the reciprocal conserved protein pairs identified for a given pair of genomes, we selected pairs randomly with replacement of as many as the number of the original pairs and repeated this procedure 100 times, resulting in 100 bootstrap AAI values for that pair of genomes. Then, 100 bootstrap AAI trees were generated and bootstrap values were calculated, defined as the occurrence of clades of the AAI tree in 100 bootstrap trees.

### In Silico MLST

The seven gene fragments of housekeeping genes *adk*, *atpA*, *dxr*, *glyA*, *recA*, *sodA*, and *tpi* were extracted from the *C. difficile* genomes by NBLAST using the sequence of allelle number 1 as the query; these query sequences were extracted from the MLST database (https://pubmlst.org/cperfringens/) collected by the University of Oxford. The best NBLAST hit with each genome was retrieved, sequences were concatenated, and a NJ tree was constructed by Muscle [32]. Redundancy was removed by deleting multiple sequences per sequence type (ST), recording the number of members per ST. For comparison, the complete genes instead of MLST fragments were also concatenated and analyzed.

### Identification of Toxin Genes by PFAM Domain Searches

Prodigal software was used to identify all protein-coding genes across all analyzed *C. difficile* genomes [33]. The Pfam domains in the proteins of these genomes were identified using HMMER 3.1b2 [34] to scan across the 16,306 profile hidden Markov Models in the Pfam database version 30.0 [35]. Presence of genes coding for toxin A or toxin B was identified on the basis of presence of the Pfam domains PF12918, PF12919, PF11713, and PF12920. CDT protein A was identified on the basis of presence of the Pfam domain PF03496 and CDT protein B by presence of PF07691 and PF03495.

### Read Coverage Analysis from Metagenomic Data

To assess if metagenomics can be used for identification of CDI, we investigated the recently published shotgun metagenomes produced from stool samples of patients with suspected or confirmed CDI in two different hospitals located in Canada [6] and Italy [33]. A total of 228 metagenomic samples were available from the Canadian study, including CDI cases and controls. We blinded our analysis, not knowing which of these samples were from controls and which were from patients. Of the 15 Italian metagenomic samples available, we only analyzed two datasets from clinically confirmed CDI cases, as diagnosed by the authors [36]. Reads were downloaded from NCBI BioProjects PRJNA297252 and PRJNA297269 at NCBI’s Sequence Read Archive (SRA) database. The SRA metagenomic data were converted into FASTQ format using ‘SRA Toolkit’ software and then aligned to the 663 *C. difficile* genomes using Burrows-Wheeler analysis (BWA) software version 0.715 [37] using default parameters. All reads were compared to *C. difficile* sequences and the *C. difficile* genome to which the most abundant reads were matched was then identified. The genome coverage of the metagenomic reads against that reference genome was determined by using the *genomecov* function in BEDTools software [38]. For four datasets that had a genome coverage of over 50%, the reads were plotted using the Integrative Genomics Viewer (IGV) [39] to illustrate the presence or absence of toxin operon genes, using the complete genome of *C. difficile* NC_009089.1 as a template. A sample with lower genome coverage was included as a control. The five sequences presented here include SRR2565933, SRR2565934, and SRR2565548 (BioProject ID PRJNA297252) and SRR2582247 and SRR2582248 (BioProject ID PRJNA297269).

## Results and Discussion

### Average Amino Acid Analysis of Taxonomic Type Strains Belonging to Firmicutes

**Fig. 1:**
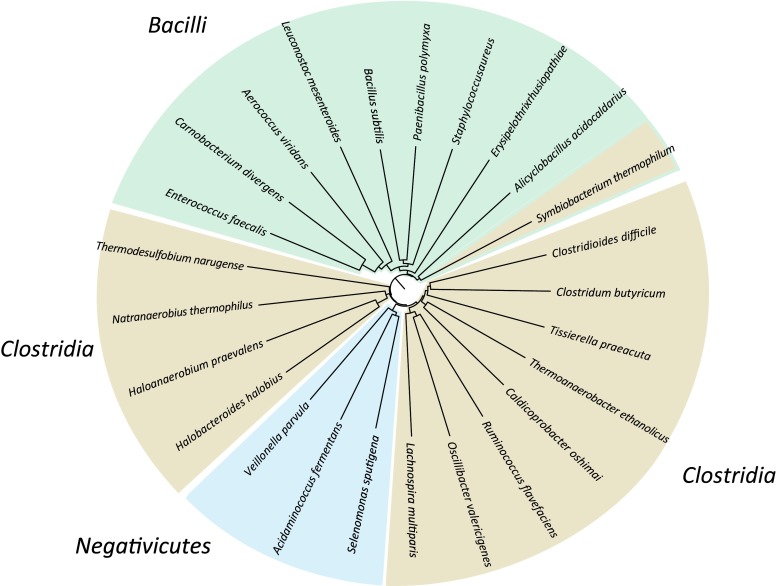
Amino acid identity (AAI) tree of 25 Firmicutes type strains. Members of the Clostridia are colored brown, Bacilli green, and Negativicutes blue

A cladogram tree was generated based on AAI analysis using 25 genomes from Firmicutes taxonomic type strains (Fig. 1**)**. All genomes cluster in their expected class, although the Clostridia class (shown in brown) is split up into two large clusters. The first Clostridia cluster starts with *C. difficile* and for the second member of this genus, *C. butyricum*, its closest neighbor is *Tissierella praeacuta*, which at first seems to be strange*.* However, the type strain of this species and that of *Clostridium hastiforme* have been shown to be identical, so that the two genus names can be considered synonyms, with priority for *T. praeacuta* [40]. The first Clostridia cluster is followed by a cluster of Negativicutes, represented by type strains of the genera *Veillonella*, *Selenomonas*, and *Acidaminococcus* (shaded blue in the figure). These Firmicutes produce negative Gram stains and, like all Gram-negatives, contain two cell membranes. Nevertheless, based on their genome content, they are distant to both well-characterized Gram negatives and Gram positives, but slightly closer to Gram-positives [41]. All Bacilli type strain representatives compared in this analysis are found together in the next cluster, indicated in green. The only exception to the expected position in the tree is the type strain of *Symbiobacterium thermophilum*, which is found as part of the Bacilli cluster although this genus is considered to belong to the Clostridia, as indicated by the brown shading in the figure. From its position in the tree, it is obvious that the type strain of *Symbiobacterium thermophilum* shares considerable characteristics with Bacilli. That is no surprise, as these organisms live in strict symbiosis with Bacilli, which has probably resulted in frequent gene swaps [42]. From Fig. 1**,** we conclude that AAI analysis is able to correctly group Firmicutes genomes according to recognized taxonomic groupings.

### Average Amino Acid Analysis of Completely Sequenced Clostridia Members

**Fig. 2:**
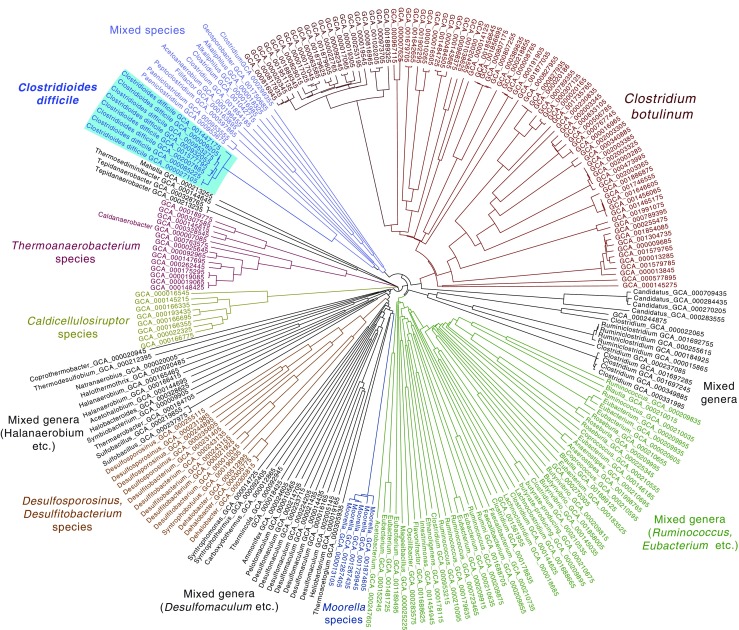
AAI tree of 234 completely sequenced genomes belonging to the Clostrida class. The light blue shading (top left) identifies the 8 included *Clostridioides difficile* genomes. For clarity, branches are colored according to their main clusters with same-color descriptions added. Branch labels only giving CGA numbers refer to genomes from strains described as ‘*Clostridium sp*.’, unless indicated otherwise with species-specific descriptions outside the tree

The next AAI analysis concentrated on completely sequenced genomes of the Clostridia only. A total of 234 genomes were compared, this time not representing a selection of type strains but all genomes that were completely sequenced at the time of analysis. In Fig. 2**,** distinct clusters are colored, and some of these nicely overlap with species, for instance the clusters of *Moorella thermoacetica*, *Caldicellulosiruptor saccharolyticus*, or the eight fully sequenced *C. difficile* genomes that were included, highlighted in bright blue. The type strain of *C. difficile* (ATCC 9689/DSM 1296) that was used in Fig. 1 is not included here, as its genome sequence is not yet complete. Compared with the diversity observed in *C. botulinum*, the eight *C. difficile* genomes are much more similar to each other and are clearly separated from the other species included here. The closest neighbor of *C. difficile* on this tree is *Paeniclostridium sordellii*. Their close relationship was also demonstrated with a 16S rRNA neighbor-joining phylogenetic tree [9]. Nevertheless, most clusters in Fig. 2 contain a mix of genera. This observation demonstrates that the taxonomic divisions within the Clostridia are still not well resolved. One reason for this may be that the phylogenetic relationship on which taxonomy is partly based is using a relatively small selection of housekeeping genes only (such as *gyrA* or *recA*) as well as 16S rRNA gene sequences. This approach works well for a number of bacterial families, where species are so clearly divided that these marker genes can reliably be used to define taxonomic divisions. In such cases, the findings obtained with analytic tools that assess the similarity of a large number of genes fit the taxonomic frame quite nicely. The AAI analysis applied here is such a multiple-gene comparison tool, and with this method, we have obtained results in good agreement with taxonomy for *Pseudomonas* species [27]. However, a number of Clostridia members presented in Fig. 2 are not completely resolved according to their taxonomic description, indicating that the currently used division is not always in accordance to their degree of genomic similarity.

### Average Amino Acid Analysis of 663 *C. difficile* Genomes

**Fig. 3:**
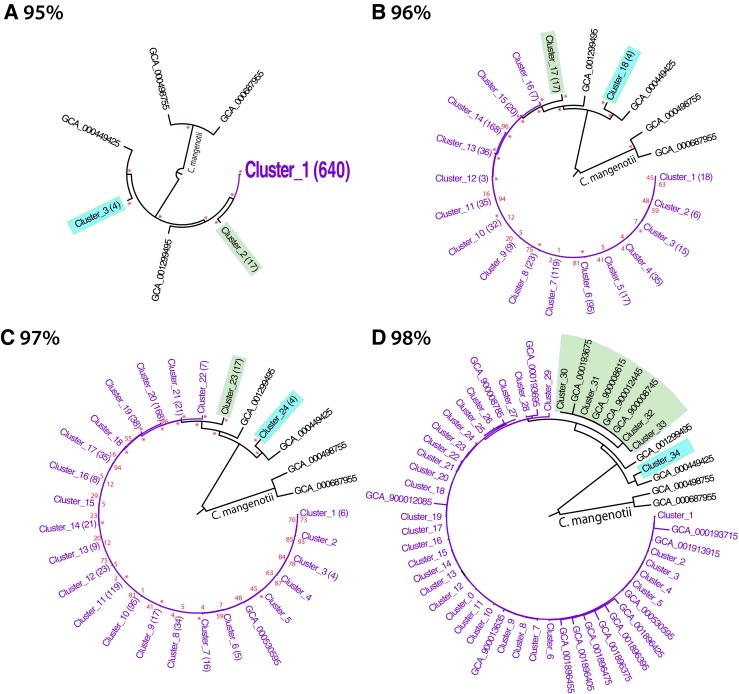
AAI trees of 663 *C. difficile* genomes collapsed at a range of cutoffs from 95% (**a**) to 98% (**d**). The number of members of a cluster is indicated between brackets (only shown for clusters containing 4 or more members, not shown in **d**). Two clusters that are discussed in the text are marked in blue and green. Numbers in red (not shown in **d**) give the confidence of nodes and clusters, with an asterisk for value 100. An unscaled version with all confidence values added is available as supplementary Fig. S1

Average nucleotide analysis (ANI) is often applied to bacterial taxonomy, whereby a cutoff of 94% is considered suitable [43, 44]. If amino acid-based AAI were to be added as an extra tool to compare strains within a species, the most suitable cutoff needs to be determined. We therefore analyzed the 663 currently available *C. difficile* sequences (complete genomes combined with those available as multiple contigs) by AAI. Ten genomes assigned to ‘*Clostridium* sp. only’ were included, and two *C. mangenotii* genomes were added for comparison as this is the closest neighbor of *C. difficile*. Figure 3 shows a compilation of trees produced with different cutoffs from the 663 *C. difficile*. At the default cutoff level that allowed ≥ 95% identity per branch, five branches were produced containing *C. difficile* genomes, two with single genomes and three representing clusters. The vast majority (640) of *C. difficile* genomes clustered together, as the default cutoff level could not distinguish these. This suggests that the vast majority of sequenced *C. difficile* genomes are relatively similar, but it also identifies 23 genomes that are more distant. This is relevant in view of the present trend to rely on in silico analysis for taxonomic assignment. The single genome branches in Fig. 3a represent one isolate for which no metadata were provided, and an isolate that had originated from a French study on *C. difficile* infections [45]. The four genomes that formed a separate cluster (colored blue in the figure) are from diverse geographic locations (two from Northern Iraq and two from the French national strain collection [13]). Likewise, the cluster comprising 17 genomes (shown in green) contained 9 isolates from a large Canadian initiative that sequenced a total of 470 strains, mixed with human isolates from Austria, Ireland, and Italy, and an equine isolate from Slovenia [46]), 2 samples supplied by a veterinary institute in Arizona for comparison in the Human Microbiome Project [47] and a 2007 isolate from a Food Safety Centre in Ireland [48]. The last genome in this cluster is from a strain described as a ‘non-epidemic human isolate’, presumably from the USA. At least one of the strains in this 17-member cluster was associated with severe diarrhea but several were described as non-pathogenic. The fact that this cluster contains strains from different continents and different host species suggests that the 95% cutoff of AAI groups allotropic strains with heterologous properties. Based on the available metadata, there is clearly a mix of virulent and non-virulent strains.

By increasing the cutoff to 96% (panel 3B), the number of branches increases to 20, of which 18 are clusters. The 17 strains that clustered together at 95% are maintained (now in cluster number 17), as is the cluster of four genomes (now cluster number 18). The remaining 640 genomes are now divided over 15 clusters, the largest one containing 168 genomes and the smallest 3. The genomes that had been derived from ‘*Clostridium* sp.’ were distributed over different clusters, so these did not form a group of their own. This trend is continued when the cutoff is increased to 97%, which maintains the clusters with 17 and 4 genomes (cluster numbers 23 and 24). However, at a level of 98%, only the 4-genome cluster remains intact, while the 17 strains are now divided over 4 small clusters and 4 single-genome branches, as shown in panel d of Fig. 3. The small clusters are only partly region-specific, for instance, three Canadian strains are combined (cluster number 32), but another cluster combines the two Irish with the Italian and an Austrian strain (cluster number 30). Continuing this analysis with a cutoff of 99% results in 92 clusters and 151 single-genome branches (results not shown).

From this analysis, we conclude that the included *Clostridium* sp. genomes can be considered to have been obtained from *C. difficile* members. Twenty-three submitted *C. difficile* genomes are clear outliers, which form two clusters and two single-genome branches. Further, with an increase of the chosen cutoff, larger clusters break up into smaller ones, and this is a continuous process.

### MLST Analysis of *C. difficile* Genomes

**Fig. 4:**
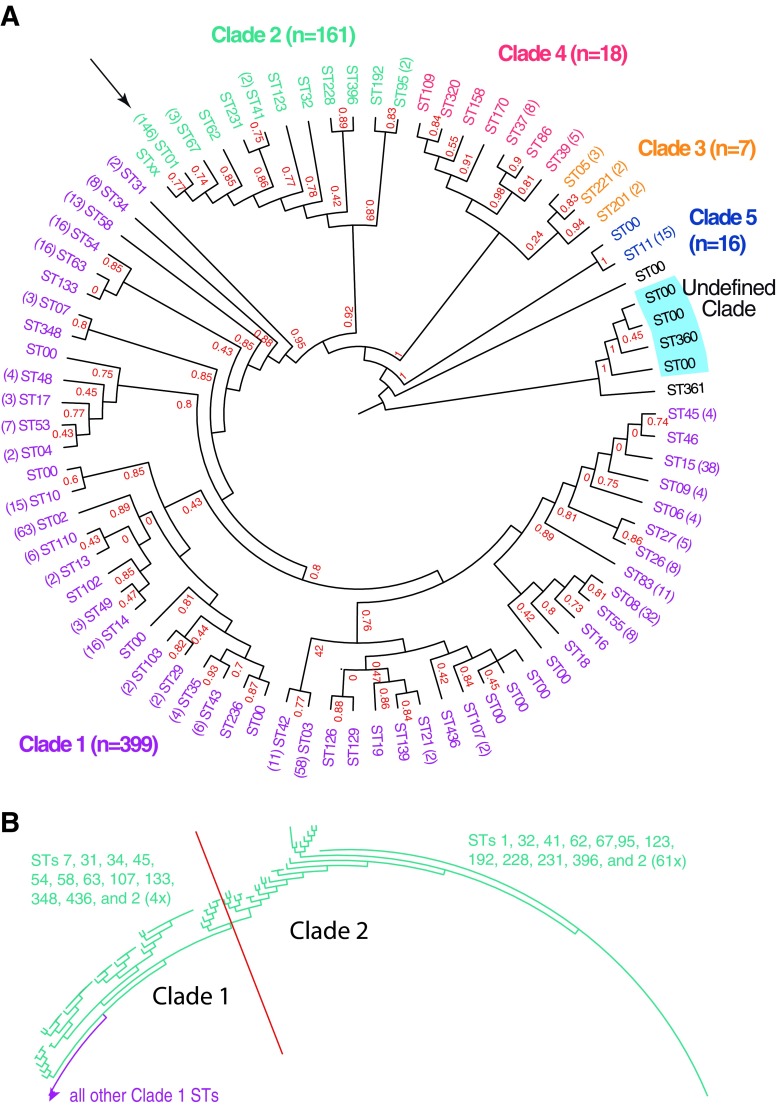
In silico MLST of 607 *C. difficile* genomes. **a** A non-redundant NJ tree with bootstrap values in red, where sequence types (ST) are given with the number of members in brackets. The arrow points to the ST01 branch with 146 members. ST00 indicates that a sequence type is not assigned to that allele combination. The four MLST clades are indicated and four genomes shaded in blue are the same that formed the four-member cluster in Fig. 3. **b** Part of an NJ tree based on complete MLST genes coding sequences. The red line separates clade 1 and clade 2 STs. A number of clade 1 STs are more similar to clade 2 sequences (thus colored green, left of the red line), and ST2 is split between the two clusters

We also analyzed the same set of *C. difficile* genomes by MLST, which is often applied to compare strains within a species [49, 50]. Complete allele fragments could be extracted from only 607 sequences. A non-redundant tree of the concatenated gene fragments is shown in Fig. 4. A table with genome accession numbers and their corresponding MLST sequence type and clade is included as Supplementary Table S1. Most of the genomes belong to MLST clades 1 (*n* = 339) and 2 (*n* = 161). The single most frequently represented sequence type is ST01 (part of clade 1) which was found for 146 genomes. Clades 3 and 4 have 7 and 18 representatives, respectively. The AAI cluster with 17 members belongs to clade 5 (for one the MLST type could not be determined). One of the two genomes that formed an outlier by AAI analysis had ST361 that grouped with four genomes in an unassigned clade; these were the four members that by AAI remained in one cluster even at 98% (Fig. 3d). The other AAI outlier was of an unassigned ST positioned between clade 5 and the unassigned clade. Supplementary Table S1 compares the STs with AAI cluster numbers at 98 and 97%. In most cases, genomes with identical ST cluster together by AAI but there are exceptions. For instance, ST1 (clade 2) is very homogeneous, but three ST1 members are placed at a large distance from their peers in the AAI trees. ST54 members (clade 1) are divided over 5 AAI clusters and include 3 single branches by that analysis, indicating that this ST is relatively heterogeneous.

Although clearly separated by MLST, the clades 1 and 2 were not clearly visible in the AAI trees of Fig. 3. We therefore re-analyzed the seven MLST genes, this time including complete coding sequences instead of the typically used MLST fragments. The result (Fig. 4b) showed that clades 1 and 2 are distinct but closely related, which explains why this division was not visible by AAI. In fact, a number of STs that are part of clade 1 are closer related to clade 2 members, based on their full-length MLST gene assessment, and four ST2 members are mixed with clade 1 STs (these 71 genomes are identified in Table S1). This illustrates that the distinction in clades based on MLST fragments is somewhat arbitrary and depends on how these fragments were chosen. Thus, the clades do not necessarily represent truly distant lineages.

### Presence of Toxin Genes in *C. difficile* Genomes

**Fig. 5:**
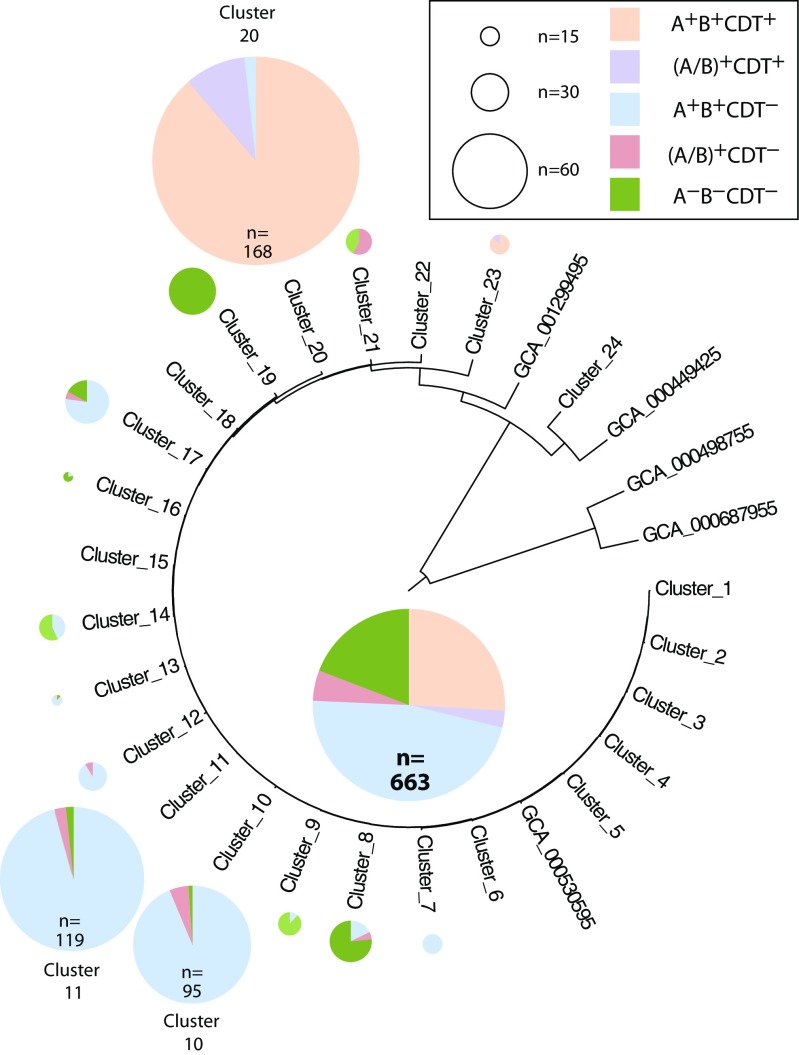
Distribution of toxin genes in the clusters of *C. difficile* collapsed at 97%. Pie charts are only shown for clusters containing 9 members or more, and those shown outside the radial tree are scaled. The distribution in all 663 genomes combined is shown inside the radial tree

Next, we assessed the presence of the *C. difficile* toxin genes, by searching for their typical Pfam domain architecture. Out of 663 genomes, 535 contained the Pfam domains indicative of presence of toxin A, toxin B, or both. This means that approximately 81% of the sequenced *C. difficile* genomes were potentially toxigenic. The two toxins cannot be distinguished based on their Pfam domains, but when two domains are found per genome, this indicates presence of two genes, presumably *tcd*A and *tcd*B. This was the case in 483 or 73% of all genomes analyzed. Most of the clusters produced at various degrees of collapse combined toxigenic strains with those lacking the tell-tale toxin Pfam domains. In Fig. 5**,** the results are presented at 98% clustering for a graphical representation. This suggests that the toxins can be present or absent independent of genetic background, which is supportive of their presumed mobility [14, 15]. Of note is that some clusters (e.g., cluster numbers 23, 24, and 25 at 98% containing 6, 3, and 38 strains, respectively) were homogeneous for lacking ToxA and ToxB, which is a striking finding giving the high incidence of these genes. Only a few of these isolates were epidemiologically related, as exemplified by the 38 strains in cluster 25, of which 20 belonged to the large strain collection from Quebec that was sequenced, but it also contained two environmental isolates from the UK, four related strains from Japan, three strains from the Pasteur Institute collection, one from Australia, one from Northern Iraq, and 7 that were sequenced by the University of Maryland. This would suggest that the cluster represents a group of globally distributed non-toxigenic isolates, though their genomes are not completely identical. At 99% collapse, these genomes are redistributed over three clusters (results not shown) though again these do not completely separate according to geographic origin. Again, these findings would suggest that a collapse at 98% is sufficient to identify closely related genomes of *C. difficile*, acknowledging that variation within the generated clusters exists, and strains from various geographic locations may be combined that share a high degree of protein content similarity.

We also searched for presence of Pfam domains indicative of the two proteins that make up CDT. Only 191 genomes contained (after translation) the Pfam domains typically present in CdtA, and two more contained CdtB domains. Interestingly, nearly all of the CDT-positive genomes also contained one or two copies of ToxA/B: 172 genomes contained ToxAB (deduced from presence of two copies of the toxin-specific Pfam domains) and 18 contained one copy. Since all genomes lacking ToxA/B also lacked CDT (with one exception), it seems the presence of these different toxins is highly overlapping. Only one genome analyzed here contained CDT but not ToxA/B (A − B − CDT+), while 310 genomes reported A + B + CDT−; the latter is most often found in the investigated genome collection. The presence of CDT in strains lacking ToxA/B has been described before [46–51], but apparently, this is rather uncommon, as we find this in only 0.15% of the strains for which a genome sequence is currently available.

### Metagenomic Analysis Can Identify *C. difficile* Infection in Stool Samples

Lastly, we analyzed metagenomic data obtained from two studies involving CDI patients. A blinded set of 228 metagenomic datasets obtained from fecal samples from Canadian CDI patients and controls [4] was screened for presence of *C. difficile* sequences. Two metagenomic samples from an Italian study [37] were also analyzed. The analysis identified two Canadian samples, C1 and C2, that produced strong coverage of *C. difficile* sequences. All other metagenomic samples from that source resulted in very poor coverage, of which C3 is presented here as it retrieved the highest number of reads of the 226 samples. The reads from the two clinically confirmed Italian cases also contained *C. difficile* sequences. Of the five samples presented here, between 0.25 and 9.05% of the total reads mapped to a *C. difficile* reference genome (Table 1). The abundance of a species in a metagenomic sample can be roughly estimated from the read coverage, so this observation indicates that *C. difficile* was present at low levels in the gut of four of these individuals. Only the stool of patient I1 seemed to have high numbers of *C. difficile* as deduced from the read coverage (Table 1). Those reads that could be mapped to a *C. difficile* genome covered between 9.69 and 98.76% of that genome. Thus, in the case of patients C1 and C2, nearly a complete *C. difficile* genome was present in the metagenomic reads. The data from the Italian study, which had resulted in approximately 10 times fewer total reads (Table 1), still covered around 52% of a *C. difficile* genome. In contrast, the reads obtained from feces of patient C3 only covered < 10% of the best matching *C. difficile* genome, although there were over 139,000 reads mapped to that species in total. The vast majority of those reads mapped to a few short specific region of a *C. difficile* genome only, which happened to be fragments of 16S and 23S rDNA and one *tRNA_met* gene (results not shown). Of note is that these RNA genes are present in multiple copies (between 13 and 15 copies of the ribosomal gene locus and about 8 copies of the *tRNA_met* gene are typically present in a *C. difficile* genome) which may explain why these sequences were picked up. These results suggest that the stool of patient C3 contained very low levels of *C. difficile*, whose presence could only be detected based on multiple gene copies of particular RNA genes. In contrast, the much better coverage of half or a nearly complete *C. difficile* genome in samples C1, C2, I1, and I2 suggests that these contained higher numbers of this pathogen.

**Table 1:** Summary statistics for each of the metagenomic datasets for the four patients with *C. difficile* infection

		Patient				
		C1 (Canada)	C2 (Canada)	C3 (Canada)	I1 (Italy)	I2 (Italy)
SRA ID of human gut metagenomic sequences		SRR2565933	SRR2565934	SRR2565548	SRR2582247	SRR2582248
Total metagenomic reads (*n*)		12,529,978	15,032,540	54,725,506	1,216,877	1,002,345
Metagenomic reads mapped to *C. difficile* (*n*, %)		148,740 1.19%	45,218 0.30%	139,248 0.25%	24,568 2.01%	90,741 9.05%
*C. difficile* strain to which the reads map best	Strain name Assembly ID origin	Strain 5.3 GCA_000586575 (Australia)	Strain VL_0181 GCA_900012755 (Canalda)	Strain VL_0083 GCA_900011925 (Canada)	Strain IT1118 GCA_001497755 (Italy)	Strain Y384 GCA_000451045 (USA)
	Genome size (Mb)	4.00932	3.97192	4.23194	4.23893	6.91120
	GC (%)	28.3%	28.6%	28.8%	28.5%	32.5%
Coverage of reads (%) on best matching *C. difficile* genome		98.76%	96.51%	9.69%	52.63%	51.04%
Presence of *tcd* genes		no	No	n.a.	yes	yes
Presence of *cdt* genes		no	No	n.a.	yes	yes

*n.a.* not applicable

**Fig. 6:**
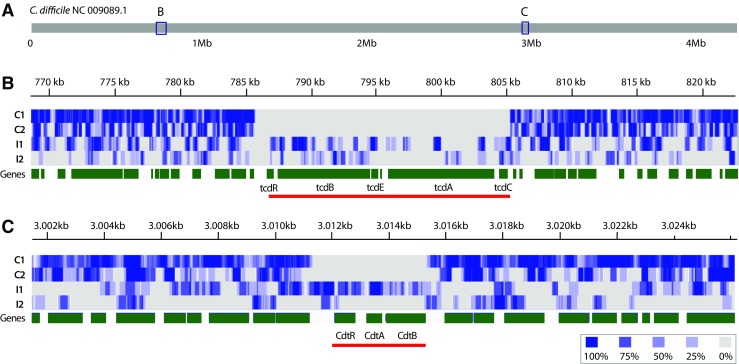
Genome coverage for the four toxin operons within *C. difficile* reference genome NC_009089.1. **a** The position of locations shown in **b** and **c** on the genome of the reference genome. **b** The read coverage of the *tcd* locus (red underlined) and its direct environment for the four metagenomic samples with heatmap colors in gray/blue as indicated. Gene locations are shown in green. **c** The *cdt* locus (red underlined) and its direct environment of the same metagenomic samples. Data taken from BioProjects PRJNA297252 and PRJNA297269

For the metagenomic sequences obtained from Canadian patient C1, the reads that matched *C. difficile* produced the best match with the genome of *C. difficile* strain 5.3, isolated in Australia [52]. For patient C2, the best match was obtained with strain VL_0181 which had been isolated from Canada. However, these two strains did not carry the *tdc* or the *cdt* operon, thus resulting in gaps in their sequence, as illustrated in Fig. 6. Indeed, *tdcA*, *tdcB*, and *cdt* sequences were absent from the metagenomic reads of C1 and C2, demonstrating that for these patients, CDI was caused by a non-toxigenic strain. In addition, our analysis shows that the two patients had been infected by different strains. Similarly, different strains were identified in the two patients from Italy. Patient I1 resulted in sequences most closely matching to *C. difficile* strain IT1118 with ribotype 018, which was responsible for outbreaks occurring in Italy, South Korea, and Japan [53], while patient I2 possessed a strain whose reads were most similar to *C. difficile* strain Y384, isolated in a hospital of Pennsylvania, USA. In these two cases, the genomes contained *tcd* and *cdt* operons, which is illustrated in Fig. 6.

In total, these data show that metagenomic analysis of stool samples can identify presence of *C. difficile*, and the degree of genome coverage can be taken as a measure for abundance of the organism. In the future, as the cost of metagenomic sequencing becomes more affordable and faster, this approach might become economically feasible for more routine analysis. We further conclude that, although patients may be diagnosed with a CDI in the same hospital, their infection was unlikely due to a common nosocomially transferred strain. More likely, endogenous, community-acquired strains, may have been responsible for these analyzed cases.

## Conclusions

The cluster analysis presented here has shown that for different members of Firmicutes, AAI clustering provides valuable insights on similarities that broadly agree with taxonomic position. At the level of genera and species within Clostridia, the clusters are less well resolved, as various genera are mixed. The taxonomic classification of *C. difficile* has encountered difficulties in the past. The WGS analysis presented here was based on AAI, which captures a large fraction of protein gene content. That clearly identified all analyzed 663 *C. difficile* members belong to a single species that is distinct from its closest relatives. At the level of strains within the *C. difficile* species, AAI analysis groups the vast majority of genomes within one cluster at 95% cutoff. This cluster subdivides as the cutoff for similarity is increased, but a clear optimal cannot be identified. Most genomes with identical STs group in AAI clusters, but there are exceptions. MLST clade 1 contains a number of STs that are more similar to members of clade 2. The analysis further showed that the toxin genes are unevenly distributed over the strains.

Metagenomic analysis of stool samples can identify cases of CDI, and CDI-causing strains can be atoxigenic. Detection of multi-copy RNA genes exclusively in metagenomic reads may be indicative of low numbers of *C. difficile* in stools. The detected sequences suggest CDI cases may be caused by different strains in patients form the same hospital. These findings support evidence for the acquisition of the pathogen within the community, with autogenous strains causing the infection. The onset of symptoms during hospitalization may be a result of treatment rather than in-hospital spread of an epidemic strain.

## Electronic supplementary materials

Supplementary Figure S1 (PDF 21 kb)

Supplementary Table S1 (XLSX 33 kb)
