# Supplementary figures and images for: Genome-Based Comparison of Clostridioides difficile: Average Amino Acid Identity Analysis of Core Genomes
Source: Microb Ecol. 2018 Feb 14;76(3):801–13. doi: 10.1007/s00248-018-1155-7 (PMC6132499; doi:10.1007/s00248-018-1155-7)

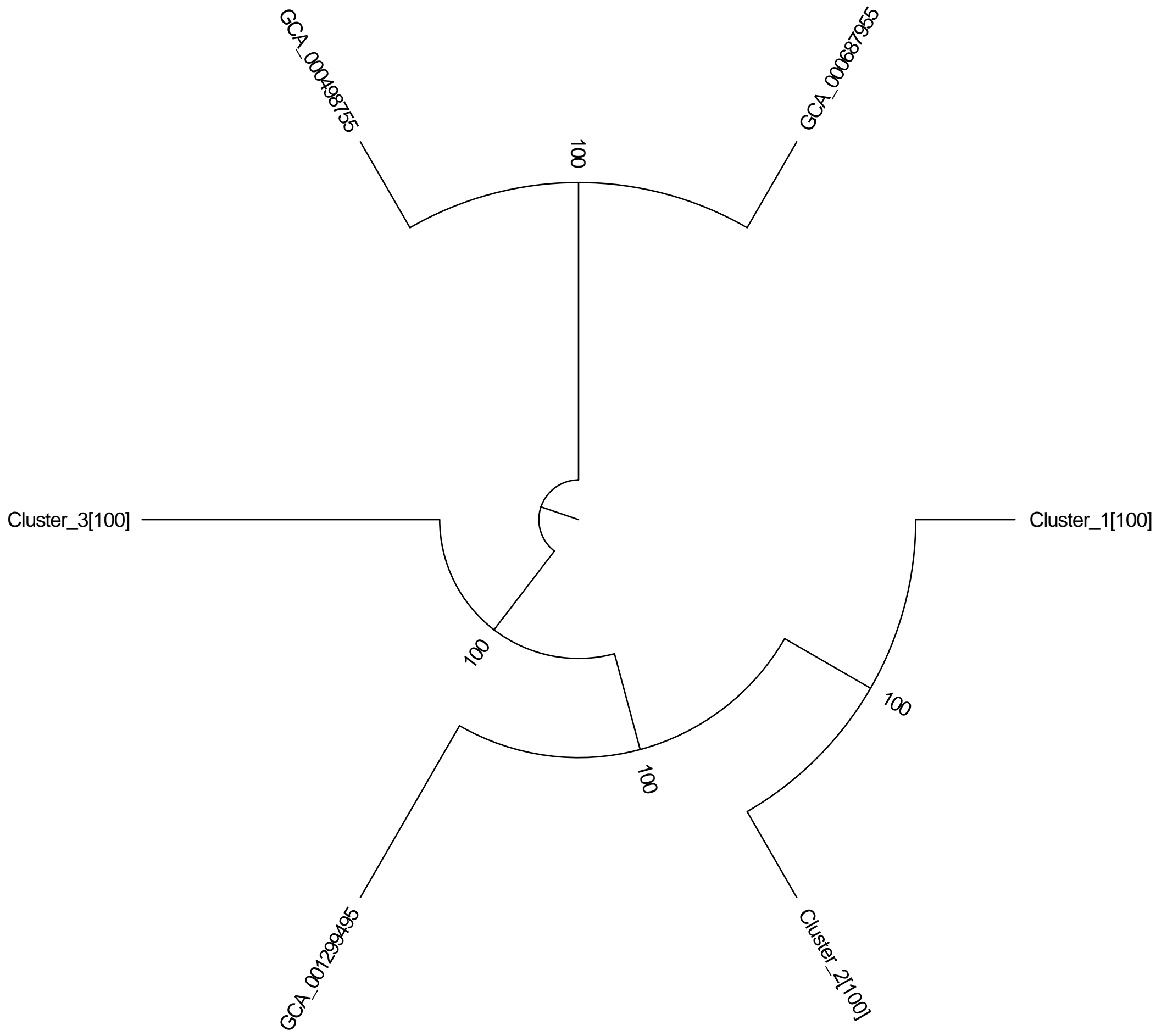

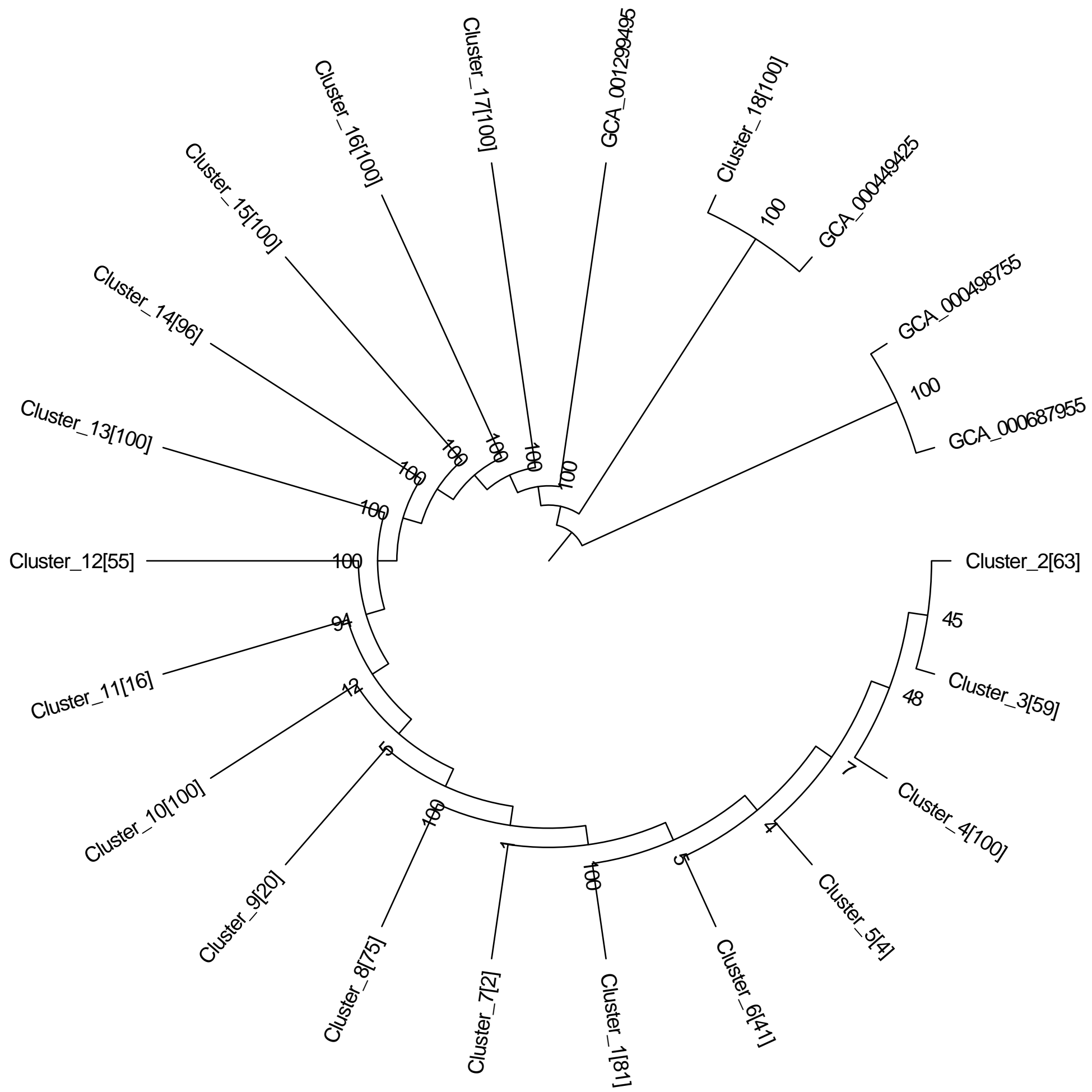

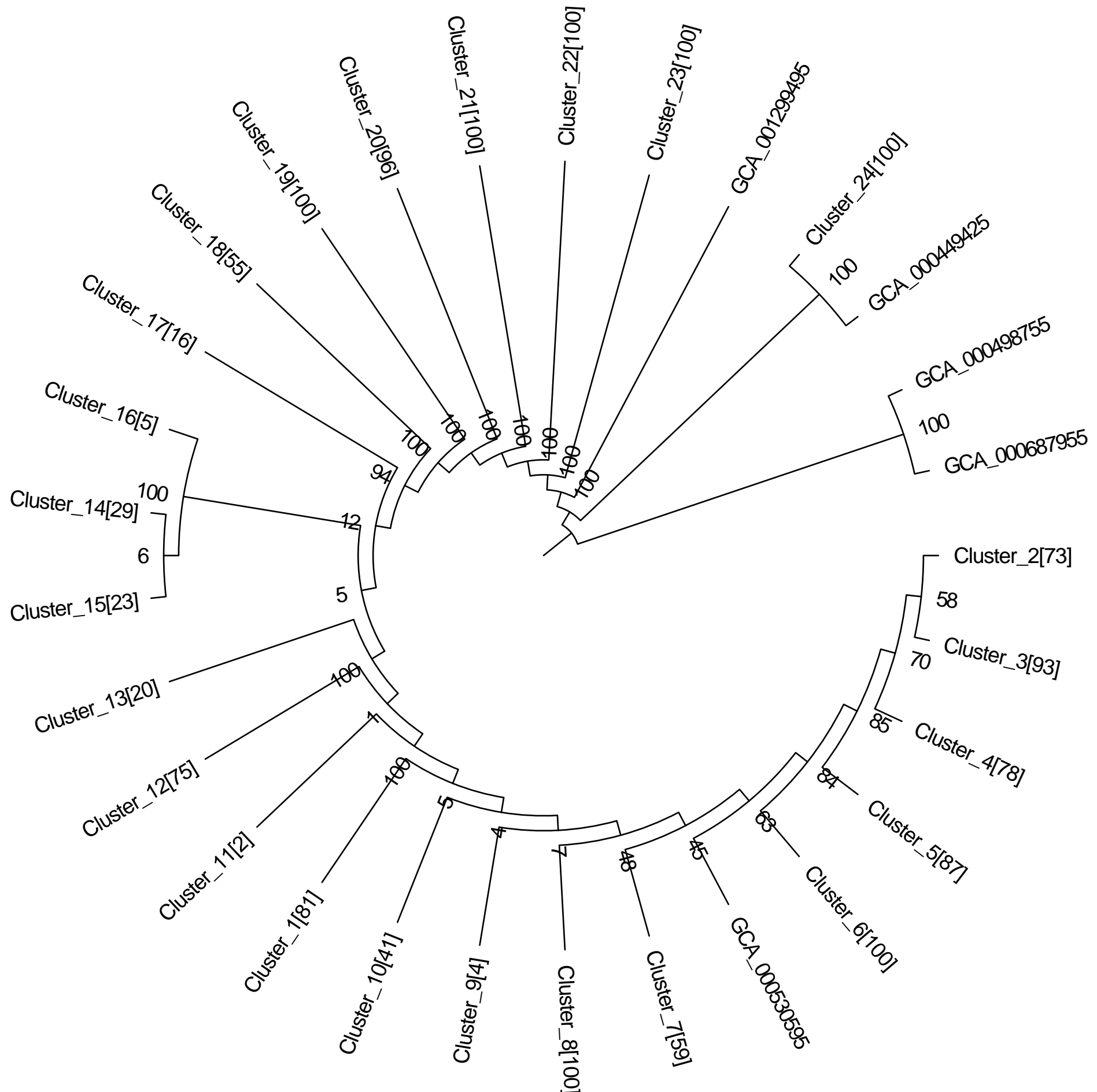

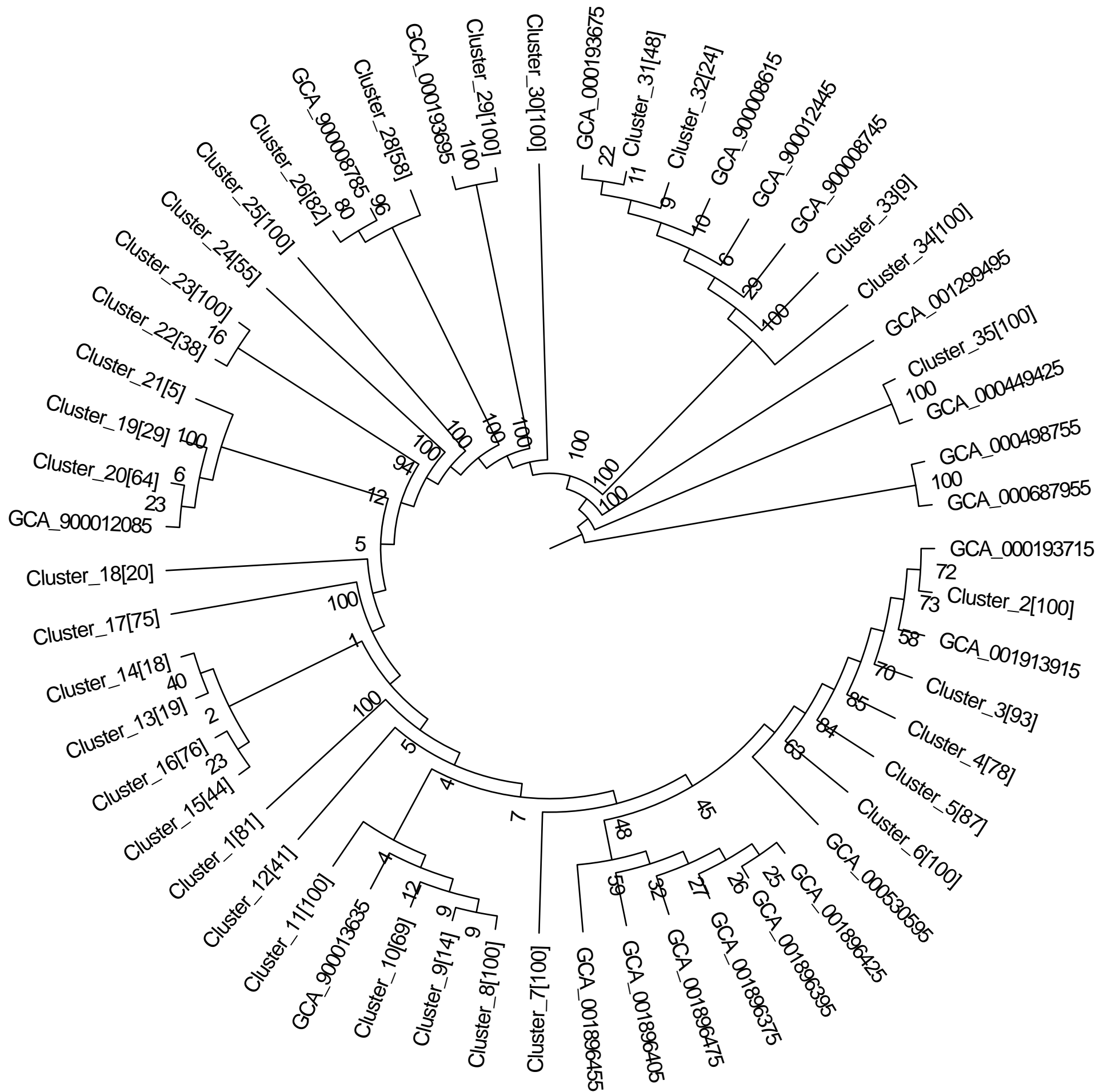

Supplement: Supplementary file 1 — (PDF 21 kb) [file 248_2018_1155_MOESM1_ESM.pdf]
